# Biomechanics of Lumbar Spine Injury in Road Barrier Collision–Finite Element Study

**DOI:** 10.3389/fbioe.2021.760498

**Published:** 2021-11-01

**Authors:** L. Pachocki, K. Daszkiewicz, P. Łuczkiewicz, W. Witkowski

**Affiliations:** ^1^ Department of Mechanics of Materials and Structures, Faculty of Civil and Environmental Engineering, Gdansk University of Technology, Gdansk, Poland; ^2^ 2nd Division of Orthopedics and Kinetic Organ Traumatology, Faculty of Medicine, Medical University of Gdansk, Gdansk, Poland

**Keywords:** car crash, numerical modeling, road safety, spine fracture, spine injury

## Abstract

Literature and field data from CIREN database have shown that lumbar spine injuries occur during car crashes. There are multiple hypotheses regarding how they occur; however, there is no biomechanical explanation for these injuries during collisions with road safety barriers (RSBs). Therefore, the objective of this study was to investigate the mechanics of vertebral fractures during car collisions with concrete RSBs. The finite element method was used for the numerical simulations. The global model of the car collision with the concrete RSB was created. The lumbar spine kinematics were extracted from the global simulation and then applied as boundary conditions to the detailed lumbar spine model. The results showed that during the collision, the occupant was elevated, and then dropped during the vehicle landing. This resulted in axial compression forces 2.6 kN with flexion bending moments 34.7 and 37.8 Nm in the L2 and L3 vertebrae. It was shown that the bending moment is the result of the longitudinal force on the eccentricity. The lumbar spine index for the L1–L5 section was 2.80, thus indicating a lumbar spine fracture. The minimum principal strain criterion of 7.4% and damage variable indicated L2 and L3 vertebrae and the inferior part of L1, as those potentially prone to fracture. This study found that lumbar spine fractures could occur as a consequence of vehicle landing during a collision with a concrete RSB mostly affecting the L1–L3 lumbar spine section. The fracture was caused by a combination of axial forces and flexion bending moments.

## 1 Introduction

Road barriers are used to prevent road injuries and fatalities. However, these barriers can cause severe or fatal injuries by transferring impact forces on vehicle occupants during crashes (Karim et al., 2012). According to the report of National Police Headquarters in Poland (2020), 1.4% of all road injuries were associated with vehicle crashes against road safety barriers (RSBs). The most serious consequences of those accidents are vertebral fractures and spinal cord injuries (Muller et al., 2014). Wang et al. (2009) revealed that front-seat occupants involved in crashes sustained spinal fractures in 12.5% of the considered cases. Adolph et al. (2013) showed that 15% of crashes with MAIS 2 + injuries included lumbar and/or thoracic spine injuries. Moreover, lumbar spine fractures occurred more frequently in late model vehicles than in early ones in frontal crashes (Pintar et al., 2012; Kaufman et al., 2013). None of the regulated or consumer information crash tests (US-NCAP, IIHS) considered lumbar spine injury as a part of their safety evaluation process, which is a cause of concern. The primary mechanism of lumbar spine fractures is caused by high-energy axial compression forces with resultant bending moments (Richards et al., 2006; Ivancic, 2013). Begeman et al. (1973) showed that the compression force was transferred from the seat pan to the lumbar spine. Munjin et al. (2011) reported that a fracture at the Th12 or L1 vertebra occurred when the patient was launched from the seat or when the patient fell back down into the seat after being launched. It is unclear how an axial compression force can act on the lumbar spine in frontal crashes. Huelke et al. (1995) hypothesized that three-point-belted occupants sustained lumbar fracture due to “submarining” of the pelvis under the lap belts. However, Tang et al. (2020) found that features that prevented submarining increased the lumbar spine forces, and as a consequence, the risk of fracture.

In previous studies, the authors demonstrated the ability to reconstruct real-world crashes using finite element method (FEM) and various types of FE human body models (HBMs) or FE models of anthropomorphic test devices (ATDs). For instance, a 50^th^ percentile male Hybrid III ATD model was used in the work of (Li et al., 2015; Tang et al., 2020). In the research by (Arun et al., 2017), they used the HBM developed by Global Human Body Model Consortium (GHBMC). However, THUMS was used by (Golman et al., 2014; Gaewsky et al., 2015; Jones et al., 2016; Ye et al., 2018), and ViVA – open source HBM was adopted by (Östh et al., 2015; Östh et al., 2016a; Östh et al., 2016b; Östh et al., 2017a; Östh et al., 2017b). The latter model was chosen for this study because it was an open source project, and it was a model of 50^th^ percentile female, for whom there was evidence that they could be more vulnerable during vehicle collisions (Pintar et al., 2012; Kaufman et al., 2013; Östh et al., 2017b).

The analysis of spine biomechanics during impacts was limited to frontal and side crash simulations in previous numerical studies. Although the Crash Injury Research and Engineering Network (CIREN) database described the spine fractures as a result of a vehicle collision with concrete barriers, the biomechanics has not been yet clarified. Because of lack of data required to simulate a specific barrier collision from CIREN database, the objective was to investigate the mechanism of vertebral fracture during a normative TB32 crash test (BSI, 2010) with a concrete road safety barrier using FEM.

## 2 Materials and Methods

### 2.1 Global Model of Vehicle Collision

**FIGURE 1:**
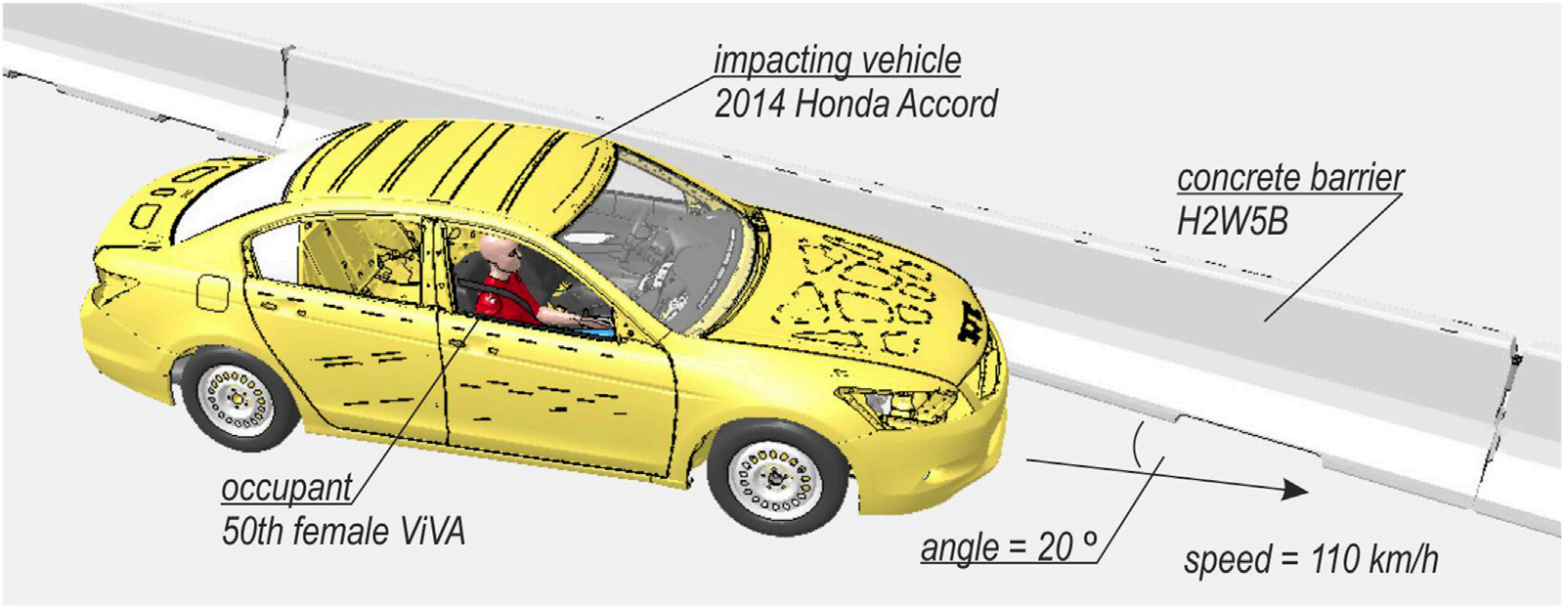
Setup of the global collision FE model.

A global FE model was created in the LS-DYNA environment (Hallquist, 2006; LSTC, 2017a). The global model consisted of a concrete RSB, an impacting vehicle, and an occupant. The setup of the global model is illustrated in Figure 1. The simulation accounts for geometric and material nonlinearities and contact effects in explicit time-integration dynamics. Because the impact angle and impact speed were difficult to infer, the TB32 crash test (BSI, 2010) was selected as a representative case (see Figure 1), i.e., a velocity of 110 km/h and an impact angle of 20°. The vehicle was positioned to hit the barrier after 0.05 s of the simulation. The selected barrier was a concrete safety system of a H2W5B class (BSI, 2010). The model of this barrier has been validated and successfully used in previous studies (Pachocki and Wilde, 2018; Pachocki and Bruski, 2020). The impacting vehicle was a 2014 Honda Accord, developed and validated by the NHTSA (Singh et al., 2018). The NHTSA’s model contained seats, seatbelts with pretensioners, and required compartment elements. It weighs approximately 1,600 kg without an occupant. For the occupant, the ViVA HBM was adopted (Östh et al., 2017a; 2016b), a 50^th^ percentile female located on the passenger side of the vehicle. The entire simulation covered 1 s of the collision. Once the global simulation was terminated, the translations and rotations of the Th12 and L5 vertebrae from the model were extracted and then imposed as boundary conditions for the detailed lumbar spine model. Specifically, the displacements were extracted from a node above Th12 and a node below L5 of the ViVA HBM. Those nodes were also used for the definition of 6-degree of freedom springs that connected adjacent vertebras.

### 2.2 Detailed Lumbar Spine Section Model

**FIGURE 2:**
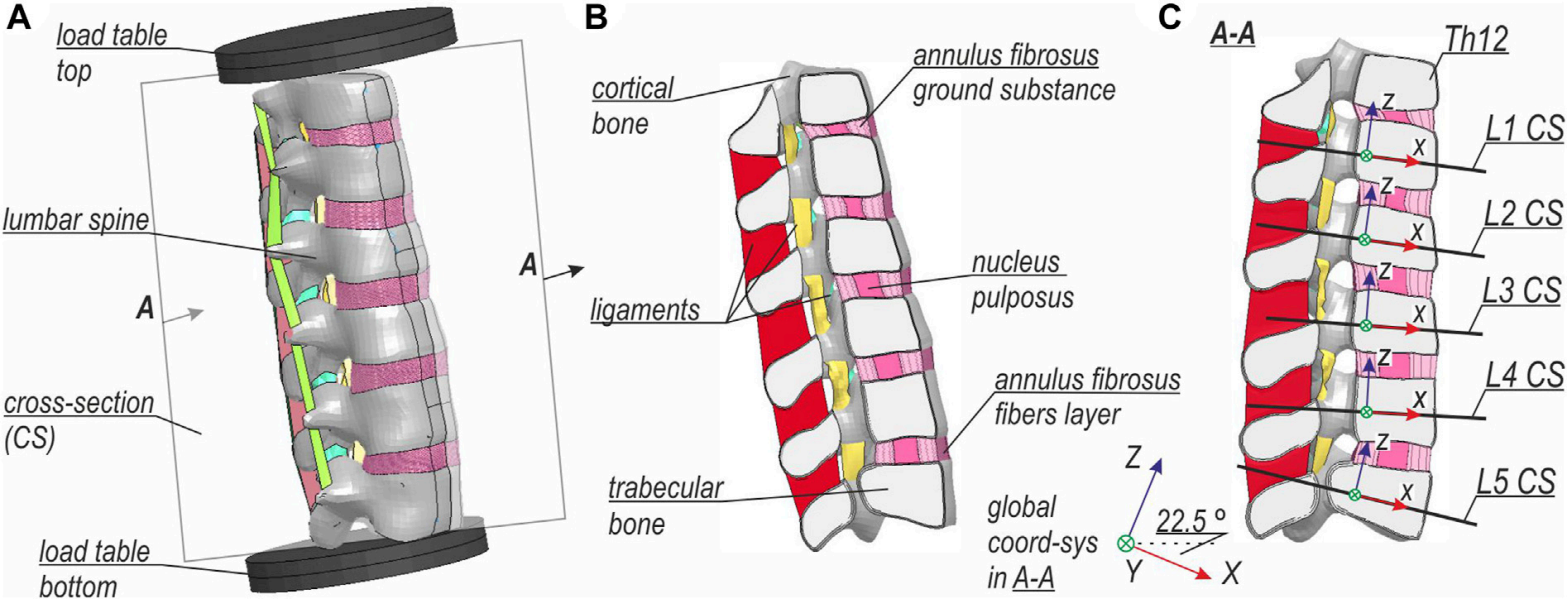
Setup of the detailed lumbar spine FE model: **(A)** general view, **(B)** half-section view, and **(C)** view on cross-sections with global and local coordinate systems.

The detailed lumbar spine model was based on the section from the 50^th^ percentile Total HUman Model for Safety v6.1 (THUMS) developed by Toyota Motor Corporation, and it was used e.g. in research by (Mendoza-Vazquez et al., 2015; Jones et al., 2016). The setup of the model is shown in Figure 2A. The comparison between the global and the local model of L-spine is provided in Supplementary Appendix A. As in the global model, a nonlinear dynamic analysis with time integration of an explicit scheme was performed. The boundary conditions from the global model were imposed on load tables that were constrained to the adjacent parts of the vertebrae: Th12 and L5. The load tables were positioned so that their centers of gravity coincided with the nodes in the global model that were used for the extraction of boundary conditions. Furthermore, the detailed model setup was rotated 25.5° in sagittal plane, which is based on the positioning of L-spine in the global model. Figure 2B presents the half-section A-A with the names of specific parts of the lumbar spine model. The internal forces in the respective vertebras were calculated in the cross-sections (CSs) located at the height of their center of gravity (CG), as shown in Figure 2C. The normal directions of the CSs planes were calculated as an average of the normal directions of the planes created on the superior and inferior endplates of the given vertebra.

**FIGURE 3:**
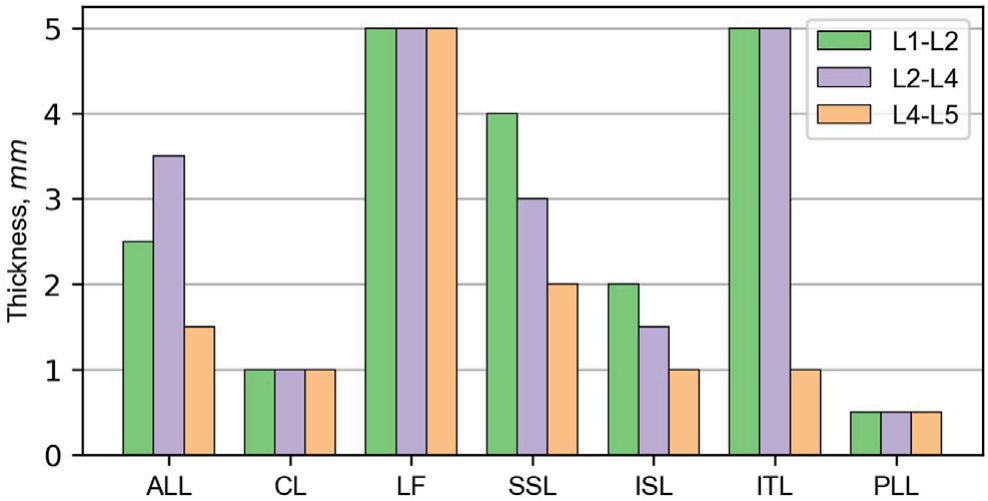
Thickness of ligaments in the lumbar spine model.

To pass several validation tests described in the works by (Demetropoulos et al., 1998; Renner et al., 2007; Xu et al., 2017) some parts and parameters of the THUMS model were modified. The FE mesh of the THUMS model was refined to a size of 1.5–2.0 mm. The detailed model consisted of 111,457 nodes comprising 37,740 shells, 503,712 solids, and 17,478 seatbelt elements (LSTC, 2017b). The properties of vertebras remained unchanged, however, the thicknesses and material properties of ligaments were modified according to the experimental data from research by (Chazal et al., 1985; Pintar et al., 1992). Figure 3 depicts the thickness of ligaments in specified sections of the L-spine. The material data for nucleus pulposus (NP) and annulus fibrosus (AF) ground substance of lumbar discs were taken from the experimental work (Schmidt et al., 2006; Schmidt et al., 2007). Additionally, NP was separated from the surrounding bones and ground substance of AF, and appropriate contact was defined. Fibers of AF were rearranged into five layers and their direction was modified to be closer to 30°. The volume content of the fibers was equal to 16% of the volume of AF’s ground substance. Their material characteristics were based on the work by Shirazi-Adl (1986). Material characteristics of soft tissues in the lumbar spine model are summarized in Table 1.

**TABLE 1:** Material properties of soft tissues in the lumbar spine model.

Soft tissue	Modulus, MPa	$\rho ,\;\frac{t}{mm^{3}}$	ν,−	Material law	References
Annulus fibrosus–ground substance	L1-L2 → $C_{1}=0.36$ ; $C_{2}=0.09$ L2-L4 → $C_{1}=0.24$ ; $C_{2}=0.06$ L4-L5 → $C_{1}=0.18$ ; $C_{2}=0.045$	1.0 e-9	0.45	Mooney-Rivlin	Schmidt et al., 2006; Schmidt et al., 2007
Annulus fibrosus–collagen fibers	nonlinear stress-strain curves	—	—	1-D nonlinear stress-strain	Shirazi-Adl (1986)
Nucleus pulposus	$C_{1}=0.12$ ; $C_{2}=0.03$	1.0 e-9	0.4999	Mooney-Rivlin	Schmidt et al., 2006; Schmidt et al., 2007
Ligaments	nonlinear stress-strain curves	1.0 e-9	0.3	orthotropic nonlinear stress-strain	Chazal et al., 1985; Pintar et al., 1992

Several criteria from the literature were applied to capture the possible fractures in the detailed model. The first was a lumbar spine index (LSI) proposed by (Ye et al., 2018). This index is based on the combined load of an axial compression force and the resultant bending moment in each vertebra of the lumbar spine. They proposed a threshold value for the L1–L4 LSI that indicated a fracture as 2.29. Another fracture criterion was based on experimental research by (Hansson et al., 1986). They described the material characteristics of a trabecular bone in the lumbar spine for compressive loads. The mean value of ultimate compressive stress equaled 1.55 ± 1.11 MPa with corresponding strain 7.4 ± 2.4%. Thus, the value of 7.4% minimum principal strain was selected as the injury criterion. The two remaining criteria of the Huber–von Mises–Hencky (HMH) effective stress and the damage variable were based on the material properties available in THUMS. For the trabecular bone, the yield stress was set as 1.8 MPa. The damage variable criterion, based on the continuum damage mechanics model, had no specific threshold assigned; thus, we proposed our own interpretation of its value.

## 3 Results

### 3.1 Global Model

**FIGURE 4:**
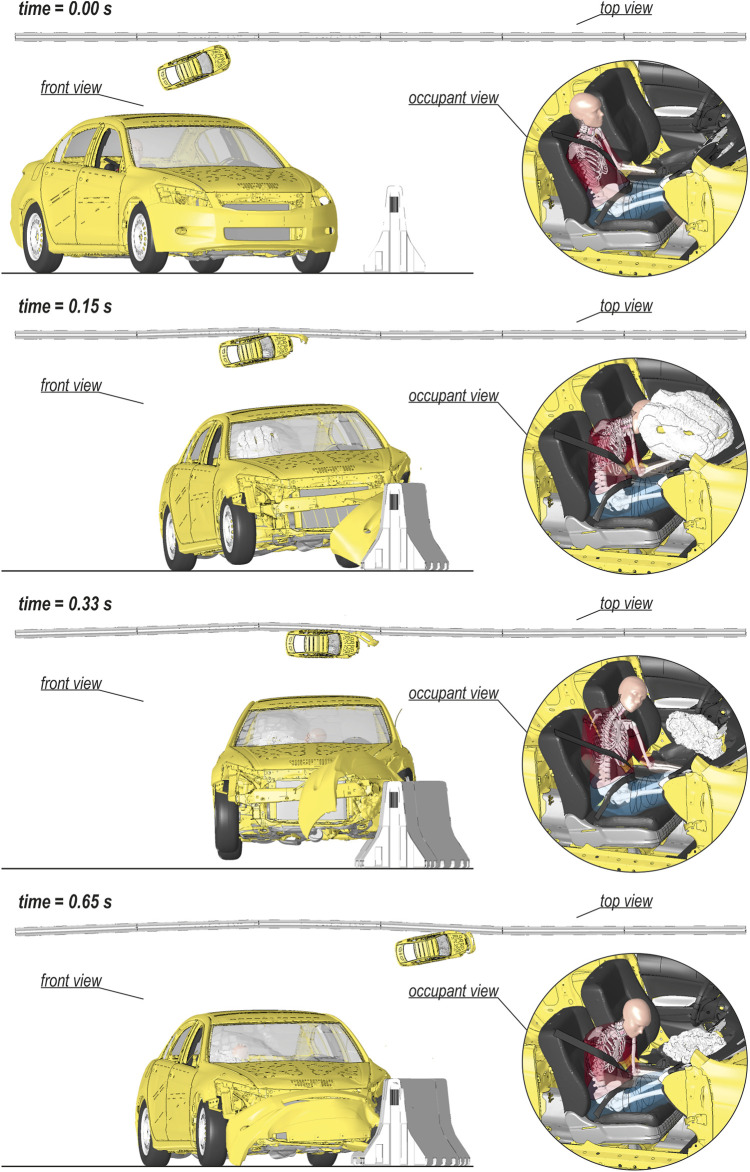
Different views on the selected time instances of the car collision with the H2W5B concrete RSB.

**FIGURE 5:**
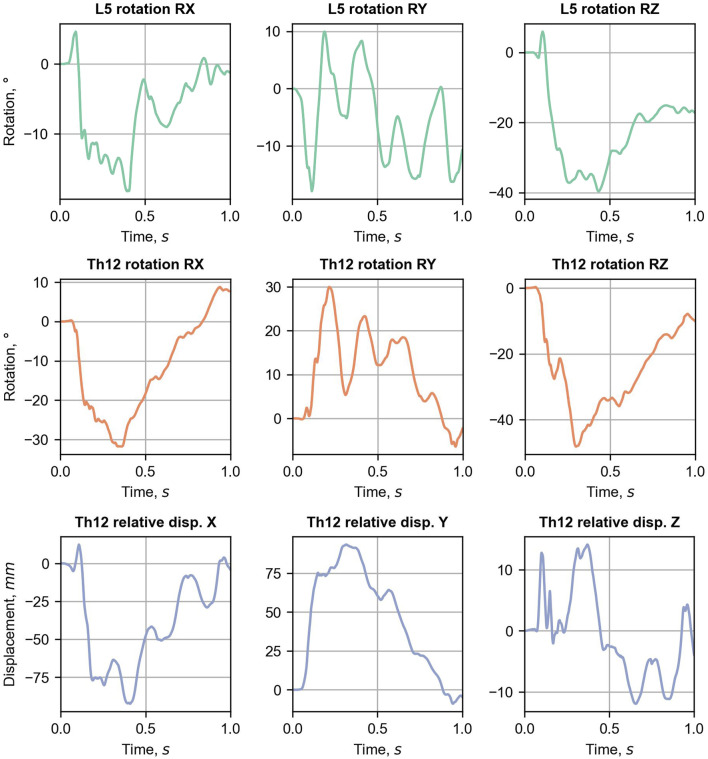
Displacement and rotation curves extracted from the global L-spine model.

The views of the vehicular crash with the H2W5B concrete RSB are presented in Figure 4 for the selected time instances. In the simulation, the vehicle hits the barrier in the connection between two segments of the barrier (0.05 s). The front left wheel of the car drove over a segment of the barrier (0.15 s), which resulted in an elevation of the entire vehicle (0.33 s). The vehicle remained in contact with the barrier, moved along the barrier, finally landed (0.65 s), and separated from the RSB (0.76 s). Owing to inertia forces, the passenger of the vehicle, after the initial impact, was forced to move forward (0.15 s). The chest and pelvis of the occupant were restrained by a three-point seatbelt system. However, because of the force vector of the impact acting in the front-left direction, the occupant bent laterally, and the shoulder belt slipped down from the upper torso. Then, the head of the passenger flexed and missed the deployed passenger airbag. While the vehicle was still elevated (0.33 s), the entire chest was placed above the shoulder belt, and the entire body of the occupant was floating over the bottom seat. The body position was maintained until the vehicle landed. Then, the upper torso wrapped above the shoulder belt, resulting in flexion of the spine, and the pelvic region dropped on the seat. The specific displacements and rotations extracted from the global model are provided in Figure 5. Those results are presented in the global coordinate system XYZ, as in Figure 2C.

### 3.2 Detailed Model

**FIGURE 6:**
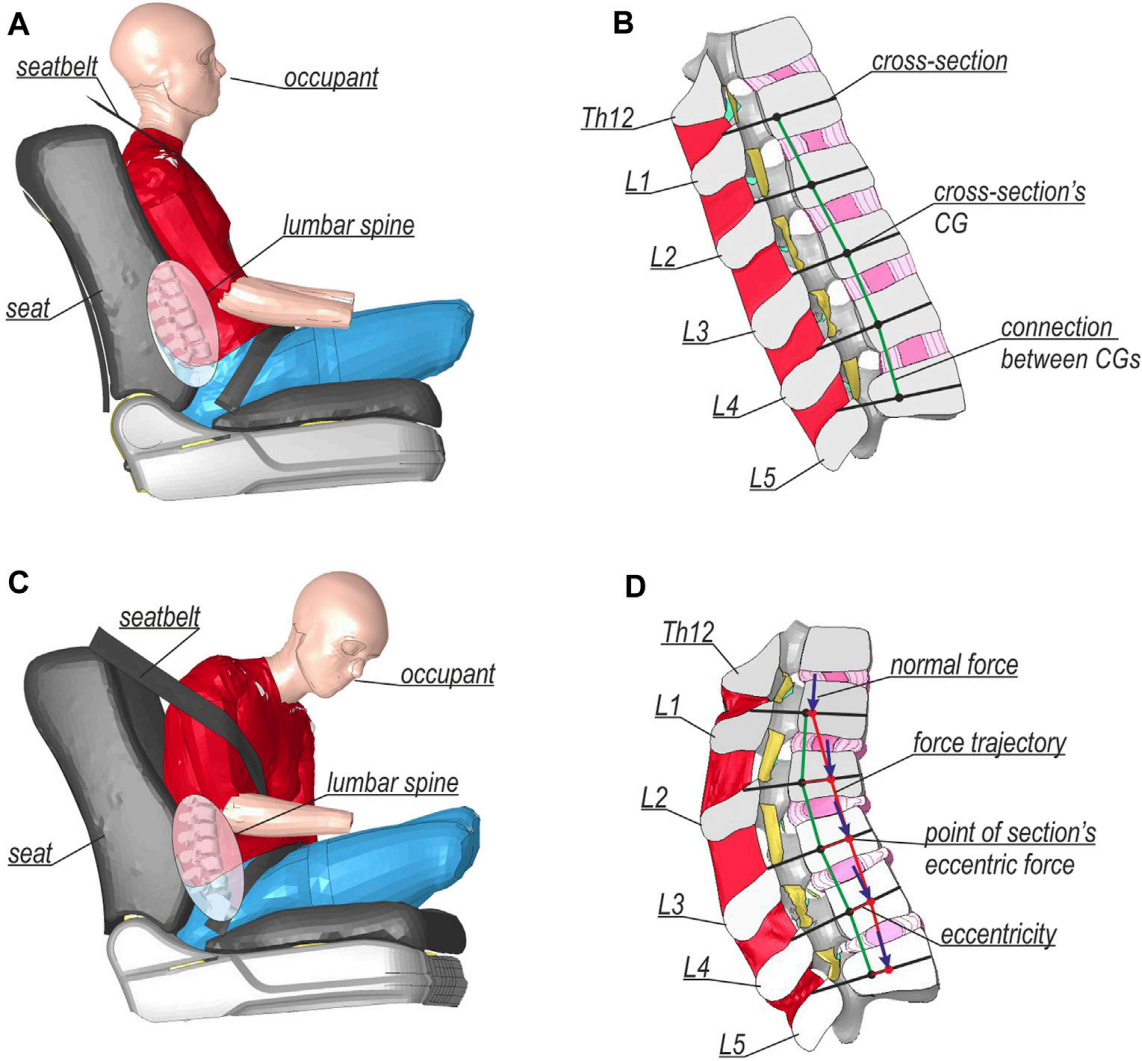
Comparison between the results for initial configurations of **(A)** global and **(C)** detailed models, and landing configurations of **(B)** global and **(D)** detailed models.

The compression force and the flexion moment were the highest internal forces in the lumbar spine. They occurred during the landing of the vehicle at 0.65 s. Consequently, a time instance of 0.65 s was selected for the analysis of the detailed model. A comparison of the passenger positions between the initial configuration and the configuration during landing is depicted in Figure 6. The hands and legs of the occupant from the global model (Figures 6A–C) were switched off for clarity, and the location of the lumbar spine section was highlighted. The detailed model results of the lumbar spine (Figures 6B–D) are presented only for the left half-section A-A. Figure 6D shows a simplified version of the trajectory of the compressive forces (denoted by the red line) during landing. The normal force and bending moments determined for each CS vertebra are listed in Table 2. Then, the longitudinal compressive force acting on the eccentricity, calculated as the bending moment divided by the normal force, was determined. For clarity, the eccentricity is shown in Figure 6D only in the local *x*-direction (see Figure 2), and it was calculated relative to the CG of the cross-section. The longitudinal force in the lumbar spine was approximately 2.6 kN, and the differences between vertebras were under 10%. The highest resultant bending moment and the greatest x-eccentricity was observed for L3. The eccentricities for each vertebra in both directions are listed in Table 2. As seen in Table 2, the moments in flexion were dominant in relation to the lateral bending moments.

**TABLE 2:** The internal forces, eccentricities and LSIs of specific vertebras during landing.

	Vertebrae				
	L1	L2	L3	L4	L5
Longitudinal force, kN	−2.44	−2.61	−2.72	−2.66	−2.59
Lateral bending moment (x-x), Nm	1.22	−8.35	−11.57	−6.21	2.54
Flexion moment (y-y), Nm	6.95	34.72	37.77	28.86	21.36
Resultant bending moment, Nm	7.05	35.71	39.50	29.52	21.51
Eccentricity (x-local), mm	2.85	13.31	13.90	10.86	8.25
Eccentricity (y-local), mm	−0.50	3.20	4.26	2.34	−0.98
LSI, -	2.07	3.03	3.22	2.89	2.60

The criterion used to estimate the risk of lumbar spine fracture was the LSI. The age-adjusted L1-L5 LSI was 2.76, and the age-adjusted L1-L4 LSI was 2.80. The threshold was 2.29; hence, the index indicated a fracture in the lumbar spine section. The LSI values for specific vertebras are presented in Table 2.

**FIGURE 7:**
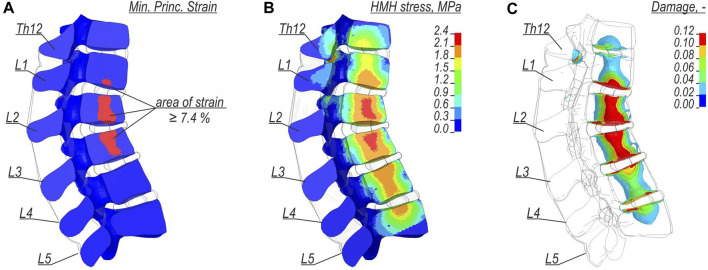
Results for the detailed lumbar spine model: **(A)** the map of minimum principal strain, **(B)** the fringe plot of HMH stresses, and **(C)** the isosurfaces of the damage variable.

The other results from the detailed lumbar spine model are presented in Figure 7 only for the trabecular bone. Figure 7A presents the map of the minimum principal strain, where the criterion of ultimate compressive strain of 7.4% was assumed. Figure 7B shows the fringe plot of effective stresses according to the HMH hypothesis, and Figure 7C shows the isosurfaces of the damage variable. The eccentric force that acted on the lumbar spine during landing resulted in kyphosis in sections Th12–L5 (see Figure 7). The largest stress and strain concentrations were found in L2 and L3 vertebras, where the highest x-eccentricities of normal forces were found. An area of high strain and HMH stress was observed in the inferior part of L1. The distribution of the damage variable indicated similar locations of potential vertebral fractures as the two previous indicators (see Figure 7C).

## 4 Discussion

This study aimed to analyze the possible injury mechanism of the lumbar spine during a car crash against concrete RSBs. FEM proved to be a useful tool for creating a complex description of a spine injury and analyzing the influence of different parameters on the risk of vertebral fracture (Fradet et al., 2014). Thus, FEA was selected to determine the physical components that appeared to be associated with lumbar spine fractures. Numerical modeling of the whole collision of the vehicle versus the concrete road barrier allowed consideration of potential risk factors indicated by other researchers, such as submarining (Huelke et al., 1995; Richards et al., 2006), belt loading on the thorax (Kaufman et al., 2013) and high-energy axial loads in the lumbar spine (Ivancic, 2013; Yoganandan et al., 2013). Apart from the global analysis of the vehicle crash, we also used a detailed model of the lumbar spine to assess the injury risk in this section. Because the essential step for the FEM is the validation of numerical results, we confirmed that our results of the concrete barrier crash test simulation were consistent with the data available in the literature (Zain and Mohammed, 2015; Pachocki and Bruski, 2020). Furthermore, the detailed model of the lumbar spine section was an improvement over the original THUMS.

Several phases of the accident can be distinguished by focusing on the occupant’s response. First, when the car hit the barrier, the passenger moved forward and was restrained by the seat belts. Subsequently, the elevated vehicle moved along the barrier. This change in the direction of the vehicle’s movement caused the occupant to lean to the left and flex over the shoulder belt. This position of the occupant during the vehicle landing might have caused the deepening of flexion of the whole spine, thereby increasing the flexion moment. Some researchers have described how axial loading with spine flexion during frontal crashes can act on the lumbar spine of a belted occupant. Begeman et al. (1973) presented experimental evidence that an axial force along the lumbar spine did exist during frontal crashes, and they hypothesized that it was transferred through a seat pan. Huelke et al. (1995) postulated that three-point belted occupants could still sustain a spinal fracture due to “submarining” of the pelvis of the occupant under the lap belt. The lumbar spine injury mechanism similar to that in the current work was described by (Munjin et al., 2011), who presented a “catapult effect”. However, Munjin et al. (2011) only indicated the influence of an axial force that was generated in the spine during the landing of the occupant. Our results showed that flexion moment could also contribute to some types of lumbar spine injuries. The described phenomenon occurred even without apparent malfunctions of vehicle interiors, as e.g. buckling of the floor or bulging of the seat that were described by (Kaufman et al., 2013).

During vehicle landing, the compression force in the lumbar spine was calculated to be approximately equal to 2.6 kN. The force acting on the eccentricities caused bending moments in the CG of the analyzed cross-section. The largest eccentricity was found at the height of vertebrae L2 and L3, which also corresponded to the highest flexion moments of 34.7 Nm and 37.8 Nm respectively. In the work by (Yoganandan et al., 2013), they proposed a fracture probability assessment based on an axial force. Authors used a drop tower tests and found a peak force of 3.7 kN that corresponded to a 50% risk of a fracture for both, thoracic and lumbar spine. In our study, the proposed method indicated a fracture probability of 11%, which led to the conclusion that some additional factors should be considered. As our research showed, flexion bending should be considered in the evaluation of injury risk as it might highly contribute to lumbar spine fractures. The combined load of an axial compression force and bending moment was investigated by (Ye et al., 2018). They proposed LSI which in our study indicated that a fracture may occur in the lumbar section; however, the index was not able to indicate the specific location of the injury. Thus, we tried other injury metrics.

In a study by (Hansson et al., 1986), based on 231 specimens, the authors described the material characteristics of trabecular bone in lumbar spines for compressive loads. The strength of Hansson’s study was a relatively large sample of simple compressive tests. Based on this research, we selected the value of 7.4% minimum principal strain as the injury criterion. This criterion indicated that the L2 and L3 vertebrae were most prone to injury. A small area of the inferior part of L1 is also marked. The distribution of HMH stress in the trabecular bone showed a large area of plastic yielding during the landing of the vehicle. The highest stress concentrations were observed in L2 and L3. This criterion also indicated a risk of yielding in other vertebrae of the lumbar spine. However, as a plasticity-based criterion, it did not immediately indicate complete failure of the material of the bone. Another criterion used was the damage variable. There are no specific usage guidelines for the damage variable for the current application. It was found that letting the damage variable equal to 10% revealed similar locations of potential injury as the strain criterion that was used.

The simulations in the current study could be associated with real-life accident cases. Querying CIREN database, accidents were filtered to cases where a vehicle impacted a concrete RSB and the belted occupant sustained a lumbar spine injury. Three cases that met these criteria were found: 100,113,783, 340,863,218, and 431,438,444. In 1^st^ case, the occupant sustained a L1 burst fracture with a major compression (>20% loss of anterior height) and disc herniation in L1–L2. In 2^nd^ case, the occupant sustained a L3 burst fracture with minor compression (≤20% loss of anterior height). In 3^rd^ case, the occupant sustained a L1 burst fracture with major compression. The locations of the potential injury obtained in the detailed model agreed with the data from the selected CIREN cases. Moreover, they were also consistent with the results available in the literature, where the authors indicated that most injuries occurred in the L1–L3 section (Begeman et al., 1973; Munjin et al., 2011; Pintar et al., 2012; Kaufman et al., 2013). Our study showed that lumbar spine injuries, most common in the frontal vehicle crashes (Pintar et al., 2012), could also occur in collisions with concrete RSBs. Moreover, to the best of the authors’ knowledge, this particular fracture mechanism was described for the first time, and it was important, e.g., in the context of the design of vehicles and road safety equipment.

The current study has its limitations as follows.

• The numerical simulation was limited to a single case study. The selected conditions were set to a TB32 crash test for a single vehicle, occupant, and RSB. However, the current approach was sufficient to explain the specific mechanism of lumbar spine injury during a car collision against the concrete RSB. The future studies should discuss the influence of impact conditions, vehicle model and passenger anatomy on the presented injury mechanism. It can be done using the methodology from e.g. Pascoletti et al., 2019a, Pascoletti et al., 2019b, where authors generated human body model, basing on the weight and height, and then using the design of experiment, they limited the number of simulations required to draw conclusion.
• The analysis of injury risk was limited to compression injuries of the lumbar spine only, because this was the injury mechanism indicated in the CIREN database. Therefore, the analysis focused only on the injuries during the landing of the vehicle; for example, a potential flexion-distraction injury from the initial impact was omitted from consideration. Further studies could investigate the potential risk of damage in other phases of collisions with RSBs.
• The detailed lumbar spine model used in this study has its limitations. Similar to THUMS v6.1, the model assumed the homogeneity of the material properties and did not describe the bone microstructure. Next, strain-rate-dependent properties were applied only to the cortical part of the vertebra. Our detailed model did not account for muscle contribution during the impact, which was justified when analyzing passenger responses. Hence, we were not able to demonstrate the specific fracture morphology, and we focused only on fracture risk assessment.

## 5 Conclusion

The current study confirmed that during a car crash with the H2W5B concrete RSB, there was a potential risk of a lumbar spine fracture at the height of vertebrae L1–L3. The fracture occurred as a consequence of a high eccentric compression force during the landing of the vehicle that was lifted by the concrete RSB. The highest eccentricity and flexion bending moments were found in vertebras L2 and L3. The largest effective stresses and minimum principal strains were also observed at L2 and L3, and the inferior part of L1. The material damage variable also indicated same location where a potential fracture could occur.

## Data Availability

The raw data supporting the conclusion of this article will be made available by the authors, without undue reservation.
