# Supplementary material for: Biomechanics of Lumbar Spine Injury in Road Barrier Collision–Finite Element Study
Source: Front Bioeng Biotechnol. 2021 Nov 1;9:760498. doi: 10.3389/fbioe.2021.760498 (PMC8591065; doi:10.3389/fbioe.2021.760498)
Supplement: Supplementary file 1 [file DataSheet1.docx]

# Appendix A


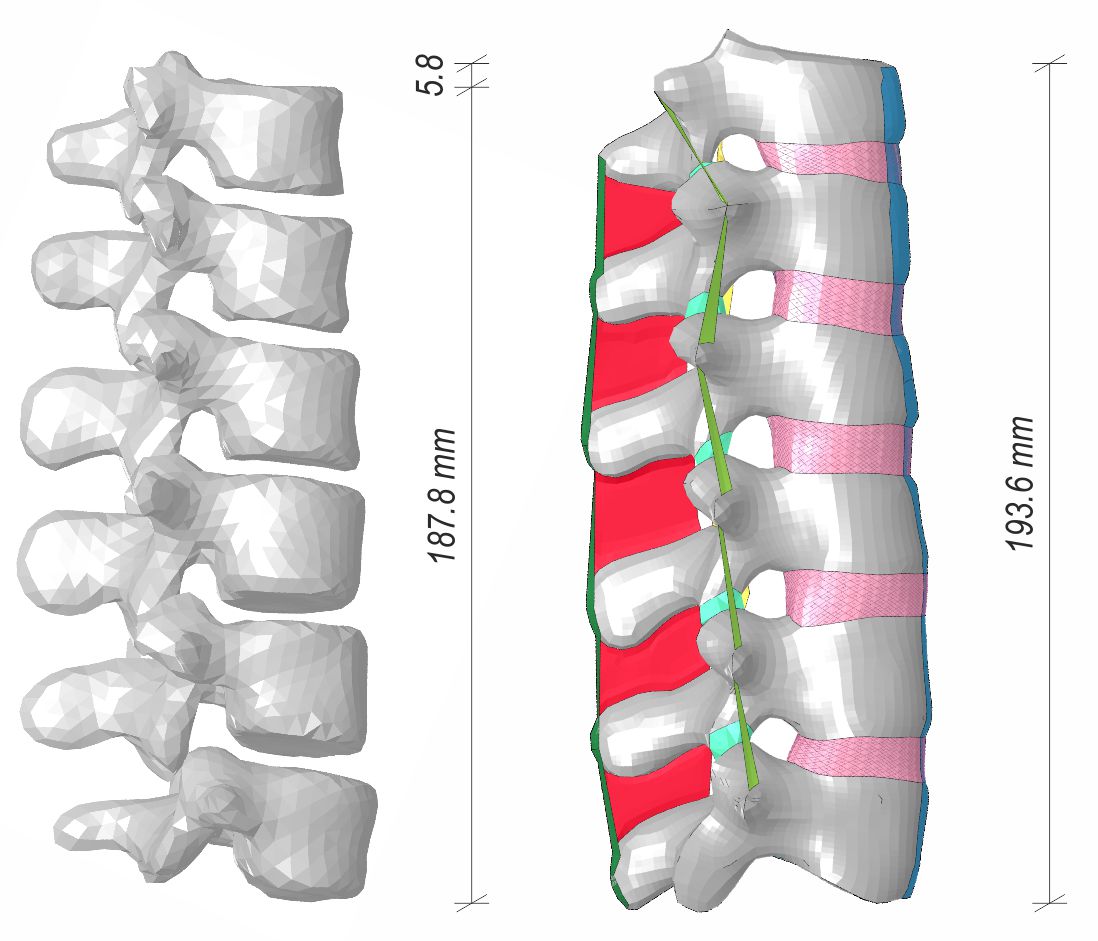


Figure A Comparison of shapes of L-spine models, the ViVA on the left and the THUMS on the right

Table A Comparison of model characteristics between the global model 50^th^ AF ViVA and the local model 50^th^ AM THUMS.

|  | Th12-L5 of 50^th^ AF ViVA | Th12-L5 of 50^th^ AM THUMS |
| --- | --- | --- |
| height, mm | 187.8 | 193.6 |
| weight, g | 886.2 | 859.1 |
| material model  of bones | rigid | nonlinear plastic |
| abdominal cavity | modelled using solids | not modelled |
| surrounding soft tissues | modelled using solids | not modelled |
| ligaments | modelled using 6 dof spring | modelled using membranes |
| intervertebral discs | modelled using 6 dof spring | modelled using solids |
